# Differential functional connectivity underlying asymmetric reward-related activity in human and nonhuman primates

**DOI:** 10.1073/pnas.2000759117

**Published:** 2020-10-29

**Authors:** Alizée Lopez-Persem, Léa Roumazeilles, Davide Folloni, Kévin Marche, Elsa F. Fouragnan, Nima Khalighinejad, Matthew F. S. Rushworth, Jérôme Sallet

**Affiliations:** ^a^Wellcome Integrative Neuroimaging Centre, Department of Experimental Psychology, University of Oxford, Oxford OX2 6GG, United Kingdom;; ^b^Frontal Function and Pathology team, Institut du Cerveau, Sorbonne Université, INSERM U 1127, CNRS UMR 7225, 75013 Paris, France;; ^c^School of Psychology, University of Plymouth, Plymouth PL4 8AA, United Kingdom;; ^d^Stem Cell and Brain Research Institute, INSERM U 1208, Université Lyon 1, 69500 Bron, France

**Keywords:** orbitofrontal cortex, reward, functional connectivity, lateralization

## Abstract

The orbitofrontal cortex (OFC) is a key brain region involved in complex cognitive functions such as reward processing and decision making. Neuroimaging studies have reported unilateral OFC response to reward-related variables; however, those studies rarely discussed this observation. Nevertheless, some lesion studies suggest that the left and right OFC contribute differently to cognitive processes. We hypothesized that the OFC asymmetrical response to reward could reflect underlying hemispherical difference in OFC functional connectivity. Using resting-state and reward-related functional MRI data from humans and from rhesus macaques, we first identified an asymmetrical response of the lateral OFC to reward in both species. Crucially, the subregion showing the highest reward-related asymmetry (RRA) overlapped with the region showing the highest functional connectivity asymmetry (FCA). Furthermore, the two types of asymmetries were found to be significantly correlated across individuals. In both species, the right lateral OFC was more connected to the default mode network compared to the left lateral OFC. Altogether, our results suggest a functional specialization of the left and right lateral OFC in primates.

## Significance

Lateralization of functions in the brain has been demonstrated in many different cognitive processes. It is supposed to increase processing abilities by reducing bilateral redundancy. Yet lateralization of reward processing, despite extremely common asymmetrical findings, has received little attention. Our neuroimaging study shows a functional lateralization of the response to reward in the lateral part of the orbitofrontal cortex (OFC), together with an asymmetric functional connectivity pattern. This particular feature was identified not only in humans but also in nonhuman primates. Our findings challenge the classical view of the OFC as a symmetrical brain region. They are urging the need for considering the specific contribution of the left and right OFC when investigating reward-related signals.

The orbitofrontal cortex (OFC) is a key brain region involved in complex behavior such as value-based decision making (1), cognitive flexibility (2), and reward-guided learning (3). This brain region is heterogeneous and can be subdivided on the basis of cytoarchitecture, connectivity, or function (4–8). The large majority of studies investigating the functional organization of the OFC consider it to be symmetrically organized between hemispheres (1, 9–12). Some unilateral lesion and stimulation studies have nevertheless shown differential behavioral effects. For instance, direct intracortical stimulation in humans revealed a left lateralization of negative experience compared to neutral experience (13). Patients with right OFC lesions were more impaired in the Iowa Gambling Task than those with left lesions (14). Asymmetrical OFC responses in healthy subjects have also been reported in functional magnetic resonance imaging (fMRI) studies (for metaanalyses, see refs. 15, 16). However, this result has rarely been discussed.

Lateralization of functions in the prefrontal cortex has been shown previously, in particular for language processing (17), visuospatial attention (18), and relational integration reasoning (15). In humans, reductions in asymmetry have been associated with impaired cognitive functions (19), and hemispheric specialization is suggested to increase processing abilities by reducing bilateral redundancy (20), indicating that there may be some benefit when homotypical areas in each hemisphere specialize. Lateralization of functions has also been reported in nonhuman primates in the context of audition and vocalization (21–24) or attention (25). Yet lateralization in other contexts, such as reward processing, has not received much attention in any species.

Using data from the Human Connectome Project (HCP) and data collected in rhesus macaques (*Macaca mulatta*), we assessed the characteristics of the asymmetrical OFC response during reward tasks. First, we identified an asymmetrical response to reward in the lateral OFC (lOFC) in both species. Second, we observed that the connectivity of the OFC with the rest of the brain was significantly different between hemispheres. Interestingly, the brain region responding differentially in the reward task was the same as the brain region showing asymmetrical whole-brain connectivity. Moreover, the two types of functional asymmetry were correlated across individuals. Finally, we found that the right lOFC was more strongly connected to the default mode network (DMN) than the left lOFC. Together, our results suggest that the left and right lOFC might support different functions, which remain to be characterized, due to an intrinsic difference in their connectivity to the rest of the brain.

## Results

### Asymmetric Reward-Related Activity in the Orbitomedial Prefrontal Cortex.

#### Humans.

**Fig. 1.**
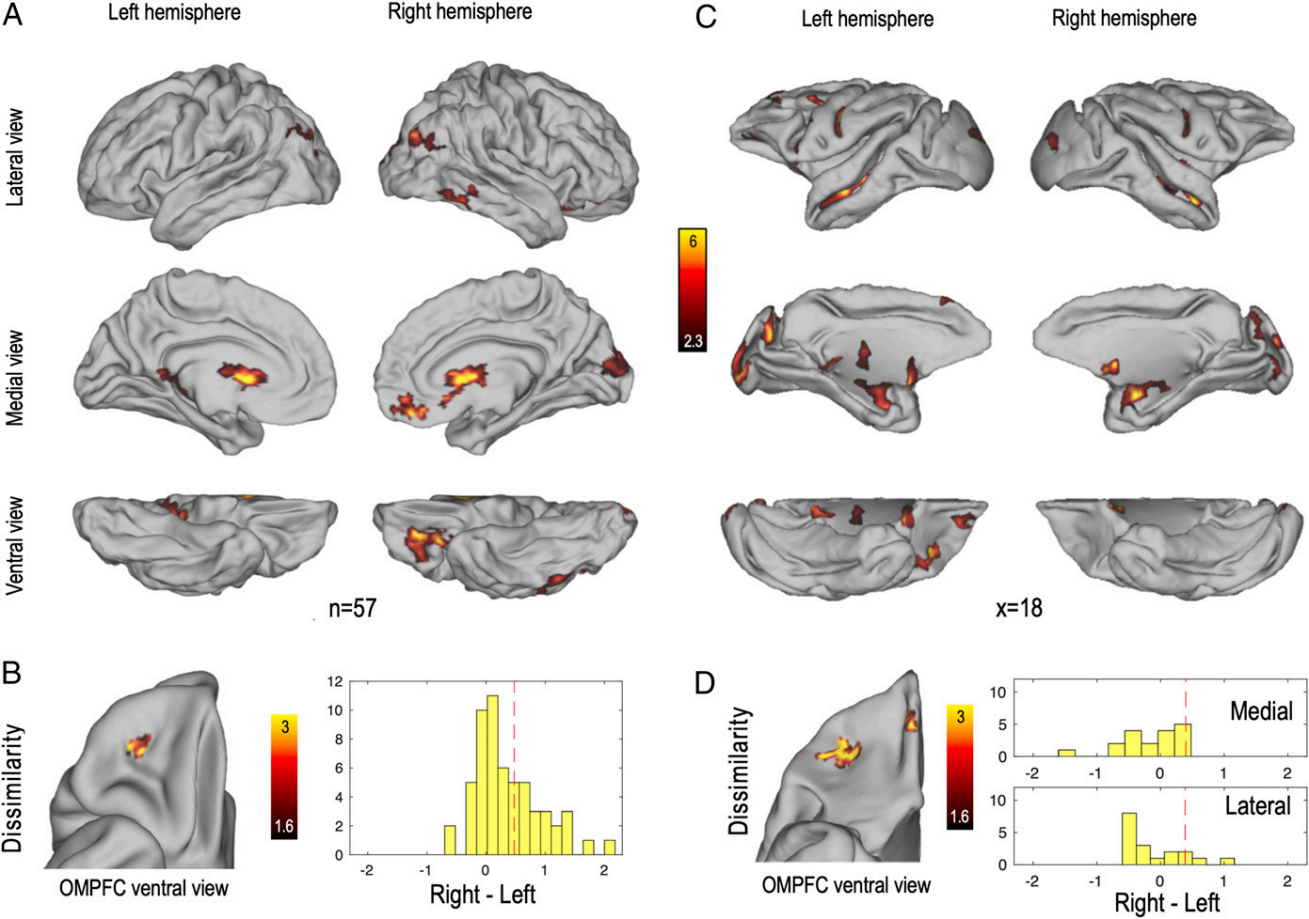
Neural responses to reward and hemispheric differences in reward responses in humans and macaques. (*A*) Statistical maps showing positive effect of the contrast reward versus punishment in humans (z > 2.3, arbitrary cluster size threshold set to 150 vertices for illustration purposes). n indicates the number of subjects. (*B*) Unsigned difference (dissimilarity) between the sizes of the effects illustrated in *A* in the left and in the right hemispheres. Color code indicates z statistics at the group level. The map is restricted to the OMPFC and cluster corrected (cluster-level *P* < 0.05, permutation tests). Results are arbitrarily displayed on the right hemisphere. Histogram represents the signed difference in reward response in the identified cluster (right minus left) across individuals. Vertical red dashed lines represent the unsigned mean difference. (*C*) Statistical maps showing positive effect of the reward regressors in macaques (z > 2.3, arbitrary cluster size threshold set to 50 vertices for illustration purposes). To facilitate species comparison, unthresholded maps are presented in *SI Appendix*, Fig. S1. x indicates the number of experimental points across protocols (*Methods*). (*D*) Unsigned difference (dissimilarity) between the sizes of the effects illustrated in *C* in the left and in the right hemispheres. Color code indicates z statistics at the group level. The map is restricted to the OMPFC and cluster corrected (cluster-level *P* < 0.05, permutation tests). Histograms represent the signed difference in reward response in the identified medial and lateral clusters (right minus left) across individuals. Vertical red dashed lines represent the unsigned mean difference.

We selected 57 subjects from the HCP for which resting-state MRI (rs-MRI) had been obtained at 7T and who participated in a gambling task designed to assess reward processing and decision-making neural responses (26). Participants had to guess whether a hidden card was higher or lower than a visible card. They received positive, neutral, or negative monetary feedback according to the correctness of the response (*Methods*). In the fMRI data, we focused on the contrast reward versus punishment to localize the reward-related activity in the whole brain (Fig. 1*A*). Replicating previous results from a larger dataset (26), this contrast also revealed higher activity for reward compared to punishment in the ventromedial prefrontal cortex (vmPFC) and in the ventral striatum. Interestingly, a significant cluster was found in the right OFC but not in the left OFC (z > 2.3, cluster corrected). Note that the uncorrected map did not reveal a response in the left OFC either (Fig. 1*A* and *SI Appendix*, Fig. S1) and that the opposite contrast, punishment versus reward, did not reveal any significant effect.

To assess whether there was an asymmetrical response to reward in the OFC, we mapped the individual z-maps onto the individual MSMAll surfaces that are registered on the symmetric MNI 152 template (27). We mirrored the data of the left hemisphere so they could be compared to the data on the right hemisphere. In order to allow the identification of asymmetries regardless of the direction of hemispheric dominance across individuals, we computed the unsigned right versus left difference in the contrast reward versus punishment for every subject and tested for a significant effect at the group level in a large orbitomedial prefrontal cortex (OMPFC) mask (*Methods*). In other words, we search for individuals with either stronger responses in the left hemisphere or stronger responses in the right hemisphere. We found a significant difference between left and right OMPFC for reward-related activity in the lOFC (p_corr_ = 1 × 10^−3^) (Fig. 1*B*). This result reveals asymmetric reward-related activity (reward-related asymmetry [RRA]) at the intersection of the lateral orbitofrontal sulcus (LOS) and transverse orbitofrontal sulcus (TOS).

To further characterize this asymmetry, we conducted a subject-by-subject analysis. We extracted the individual signed RRA coefficients from the cluster identified in the previous analysis. In humans, we observed that the right hemisphere more often showed stronger reward responses than the left hemisphere [*t* (56) = 4.99, *P* = 6 × 10^−6^; Fig. 1*B*], confirming the observed asymmetry in the whole-brain reward contrast (Fig. 1*A*).

#### Macaques.

RRA in macaques was investigated in fMRI data collected from previous studies (*Methods*). Eight monkeys who performed different types of reward-related tasks were included in the analyses. Due to the diversity of paradigms and contrasts centered on the decision and feedback periods, we used the fMRI results as a localizer to identify reward-sensitive regions rather than specific aspects of reward processing. For each monkey, we used the reward-related contrasts (*Methods*) of each session and averaged them across sessions and individuals to obtain a whole-brain map of reward-related activity (Fig. 1*C* and *SI Appendix*, Fig. S1). As in human participants, we projected each contrast map (18 experimental points; *Methods*) to a common surface and computed the unsigned right versus left difference in all available contrasts. We found two significant clusters of RRA in the OMPFC. First, we observed asymmetric reward-related activity close to the medial orbital sulcus (p_corr_ = 0.042). Second, we also identified a cluster close to the LOS, just posterior to its intersection with the TOS (p_corr_ = 8 × 10^−3^).

This second area of asymmetry in macaques lies in a similar location with respect to sulcal landmarks in the two species (Fig. 1*D*). In humans, it corresponds to the caudal part of area 11l, extending into area a47r according to the parcellation of Glasser et al. (28). This location corresponds to the caudal part of 47/12m in both humans and macaques in the standard cytoarchitectonic framework proposed by Mackey and Petrides (29).

To investigate whether there was a hemispheric dominance in the identified RRA across individuals and experimental paradigms (experimental points), we extracted the individual signed RRA coefficients from the clusters identified in the previous analysis. In macaques, the pattern was less clear than the one observed in humans (Fig. 1*D*). No significant hemispheric dominance was observed in either the medial cluster [*t* (17) = −1.03, *P* = 0.32] or the lateral cluster [*t* (17) = −0.78, *P* = 0.44]. In other words, the observed asymmetry was equally driven by experimental points with a left hemispheric dominance and experimental points with a right hemispheric dominance. Due to the diversity of task used with macaques, we were able to conduct the investigation of the signed RRA at the level of protocols. It revealed significant effects with different directions in the lOFC cluster [protocol 1: right dominance, *t* (39) = 3.12, *P* = 3 × 10^−4^; protocols 2 and 3: left dominance, *t* (83) = −2.51, *P* = 0.014; protocol 4: left dominance, *t* (75) = −2.71, *P* = 8 × 10^−3^; *SI Appendix*, Fig. S2]. This suggests that the absence of significant direction at the metaanalysis level comes from the diversity of experimental paradigms used.

#### Asymmetric functional connectivity in the OMPFC.

**Fig. 2.**
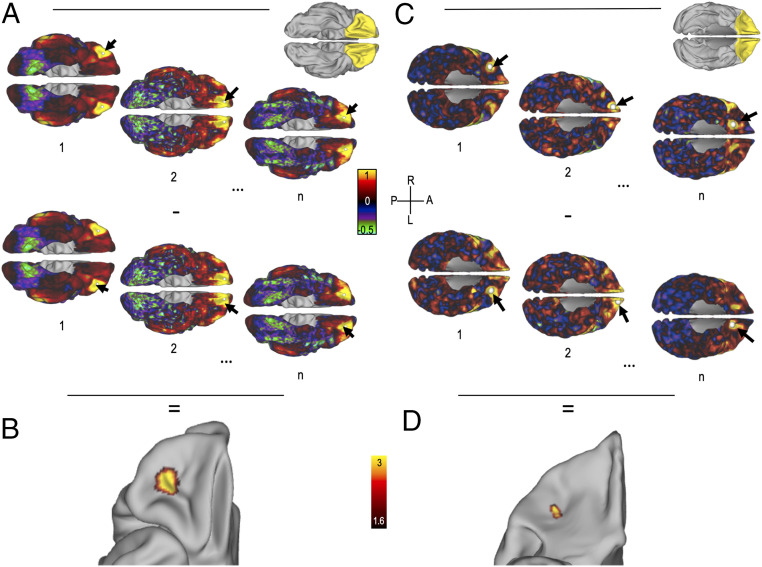
FCA in the human and macaque OMPFC. (*A*) Schematic representation of the method to compute FCA in humans. (*Top*) Functional connectivity of each vertex in the right OMPFC with all vertices in the brain. (*Bottom*) Functional connectivity of each vertex in the left OMPFC with all vertices in the brain is computed. Arrows represent the location of seeds. n is the number of vertices in the OMPFC mask (*Inset* with OMPFC in yellow). Colors indicate correlation coefficient between time series of the seed and time series of each other vertex. (*B*) Statistical FCA map (T values of the averaged unsigned difference between *A*, *Top*, and *A*, *Bottom*) displayed on the right hemisphere. Hot colors indicate high asymmetry in functional connectivity. Map is thresholded at z > 1.6. (*C* and *D*) The same as *A* and *B* but for macaque data. A, L, R, and P correspond to anterior, left, right, and posterior, respectively.

To determine whether the RRA could be linked to an asymmetry in the functional connectivity of the OMPFC, we compared the connectivity profiles of the left and right OMPFC. In both humans (*n* = 57) and macaques (*n* = 14), for each vertex of the OMPFC, we extracted the connectivity (correlation coefficients between time series) with all vertices in the brain, from the group-level time series dataset (computed with MIGP; *Methods*). Then, we computed the averaged unsigned difference between the connectivity of each pair of vertices from the left and right OMPFC (*Methods* and Fig. 2*A*). This measure (functional connectivity asymmetry [FCA]) allows us to detect dissimilarities in the pattern of functional connectivity with the rest of the brain even if the difference manifests in different left–right direction in different individuals. The resulting map is depicted in Fig. 2*B*.

We found a cluster in the lOFC with a particularly high asymmetry in humans [*t* (56) = 11.39, *P* = 5 × 10^−17^]. The same analysis conducted in macaque data revealed a very similar result, with a single cluster in the lOFC, significant at the group level [*t* (13) = 3.81, *P* = 2 × 10^−3^; Fig. 2 *C* and *D*]. Together, those results suggest that the left and right lOFC in humans and macaques are differently connected to the rest of the brain. Note that on average, the right lOFC in macaque was also more strongly connected to the rest of the brain than the left lOFC (*SI Appendix*).

In the next sections, we will 1) assess whether this dissimilarity is linked to the RRA of the OFC and 2) characterize the connectivity profiles of the left and right lOFC.

### A Hot Spot of Asymmetry in the OFC.

#### Overlap between reward-related cluster and functional connectivity cluster.

**Fig. 3.**
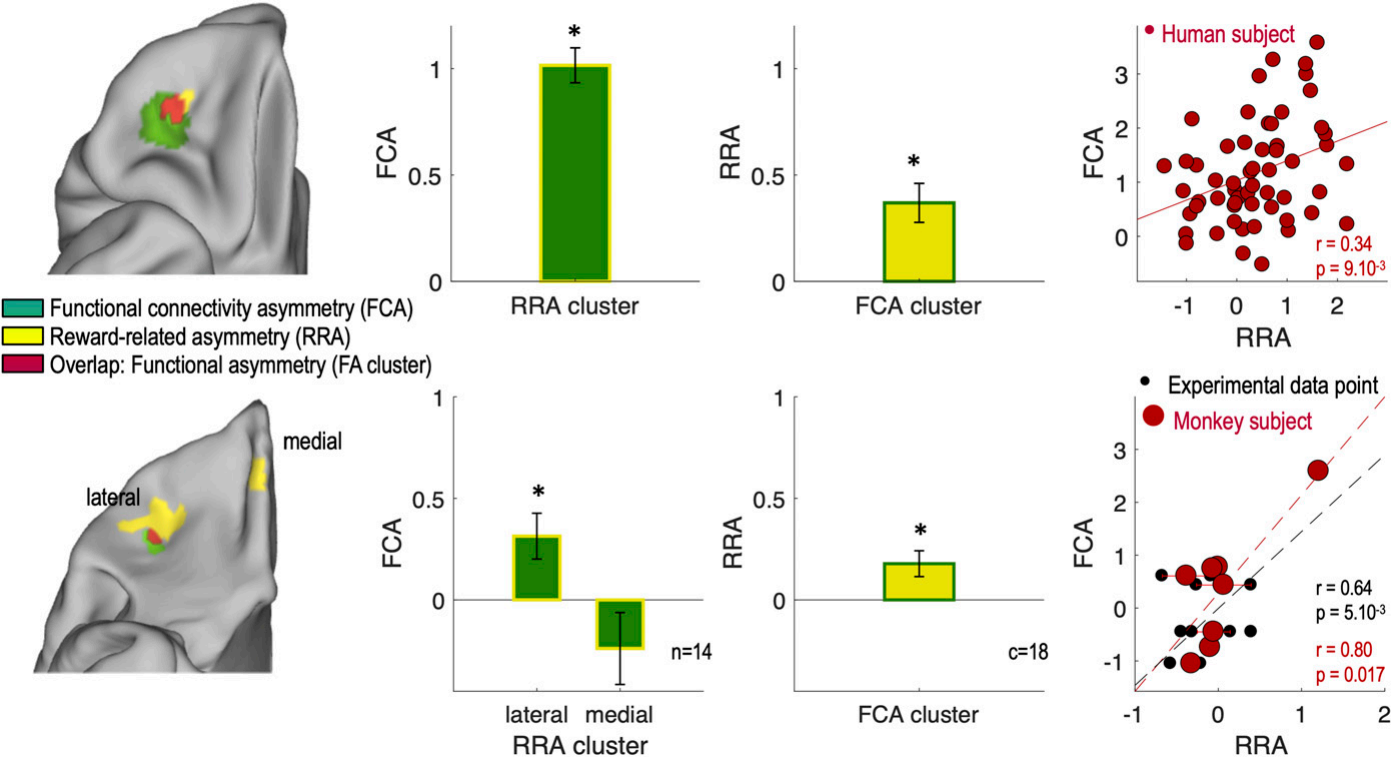
Functional asymmetry in the human and macaque OFC. The first column shows overlap (red) between the clusters of asymmetry identified in the functional connectivity (FCA; green) and in the reward response (RRA; yellow) analyses on the OFC surface in humans (*Top*) and in macaques (*Bottom*). The second column shows mean FCA coefficient across individuals in the RRA clusters (yellow). The third column shows mean RRA coefficient across individuals (reward contrasts for monkeys) in the FCA cluster (green). The fourth column shows individual participants’ FCA coefficients plotted as a function of their RRA coefficients in the FA cluster (red). (*Top*) Each red point represents one individual. (*Bottom*) Each red dot represents one monkey, and each black point corresponds to an experimental data point (from 1 to 4 per monkey). Bar plot and error bars represent mean and SEM. Stars indicate significance against 0. n indicates the number of macaques, and c indicates the number of contrasts.

To examine the link between RRA and FCA, we projected the results from the two previous sets of analyses onto a common surface (Fig. 3). We observed partial overlap of the two clusters in the lOFC, in both humans and macaques, indicating unique hot spots of functional asymmetry, as defined by both reward-related activity and functional connectivity, in the lOFC in both species. We computed the coefficient of FCA in the RRA lateral clusters and found that it was significantly higher in the lOFC cluster than in the rest of the OMPFC [humans, *t* (56) = 12.39, *P* = 1 × 10^−17^; 14 macaques with rs-MRI, *t* (13) = 2.78, *P* = 0.016; medial cluster, *t* (13) = −1.35, *P* = 0.20]. The reverse analysis, i.e., the investigation of the response difference to reward-related activity in the FCA cluster, also revealed a significant difference in response to reward between the left and right lOFC [humans, *t* (56) = 4.02, *P* = 2 × 10^−4^; macaque experimental points, *t* (17) = 2.80, *P* = 0.012].

Moreover, we extracted the individual participants’ RRA and FCA coefficients from the lOFC cluster resulting from the conjunction of the two asymmetry analyses (labeled functional asymmetry cluster or FA cluster). We found that the two measures of asymmetry, based on RRA and FCA, were strongly correlated in humans (*r* = 0.34, *P* = 9 × 10^−3^). In macaques, we found a significant correlation between RRA and FCA measures (across 18 experimental points, *r* = 0.64, *P* = 5 × 10^−3^; across 8 individuals, *r* = 0.80, *P* = 0.017). Together, these results suggest that asymmetry in functional connectivity might explain asymmetry of results in task-related activity in both species.

#### Functional connectivity characteristics.

**Fig. 4.**
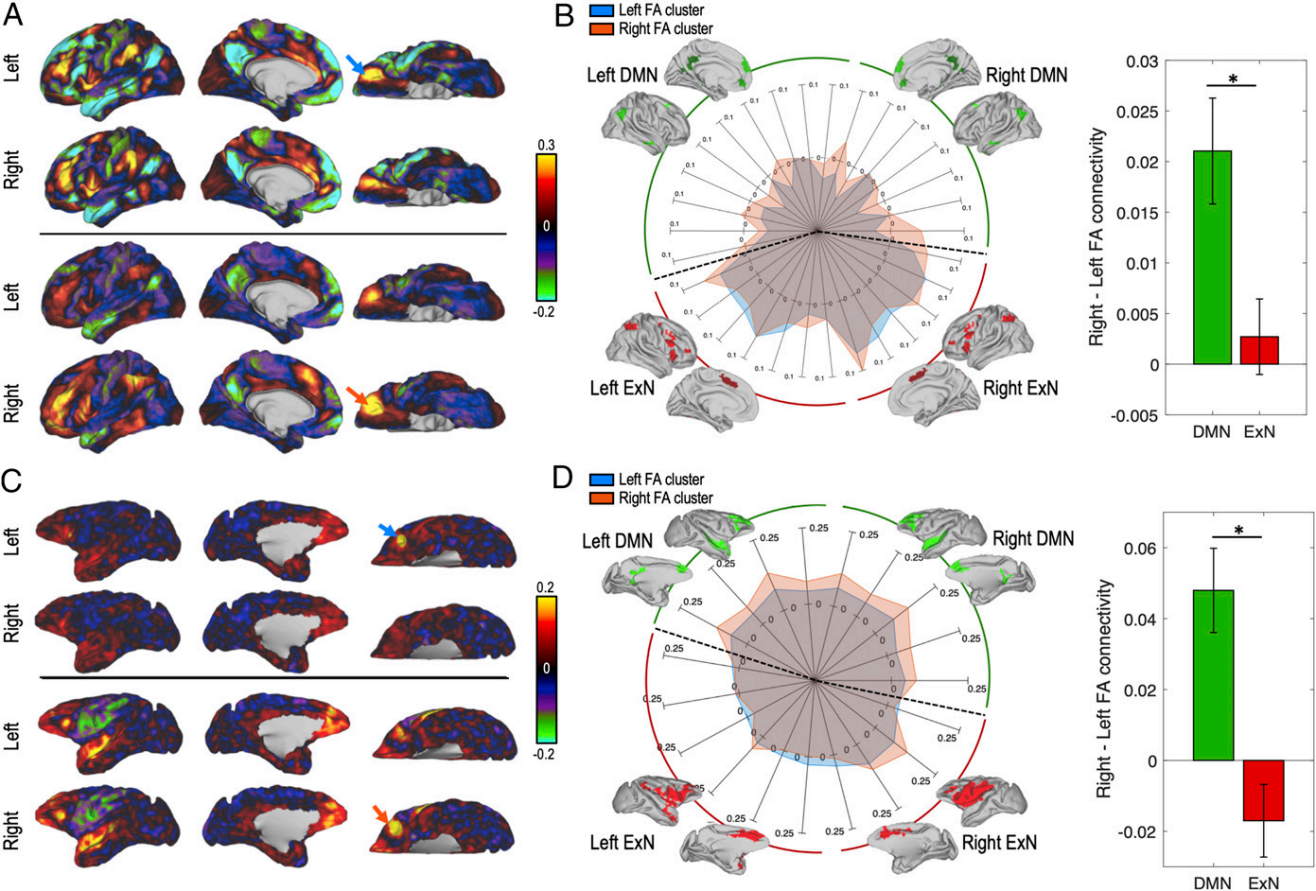
Functional connectivity of the left and right FA clusters. (*A*) (*Top*) Functional connectivity map of the left FA cluster (blue arrow) with the left hemisphere (first row) and the right hemisphere (second row). (*A*) (*Bottom*) Same as *Top* but for the right FA cluster (orange arrow). Colors indicate the strength of functional connectivity (correlation coefficients). (*B*) Connectivity profile (spider plot) of the left (blue) and right (orange) FA cluster with the DMN (green) and the ExN (red). The two networks are decomposed into several subregions that we grouped under the labels “left” or “right”; i.e., left DMN corresponds to areas belonging the DMN and located in the left hemisphere. Intensities correspond to the coupling of each seed with each target. Bar plots illustrate the significant interaction between seed and network. Bars (error bars) correspond to the mean (SEM) of the difference in connectivity of the left and right seed to the DMN (green) and the ExN (red). Stars indicate significant difference (*P* < 0.05). *C* and *D* are the same figures as *A* and *B* but for macaque data.

Finally, to further describe how the left and right lOFC are differently connected to the rest of the brain, we compared the functional connectivity of the left and right FA cluster with the whole brain. In humans, the whole-brain functional connectivity maps suggested that the left FA cluster shows a negative functional connectivity with a network including anterior cingulate cortex, posterior cingulate cortex, and temporoparietal junction. We will refer to this network as the DMN. We also observed that both seeds were positively connected to a frontoparietal network, which we refer to as the executive network (ExN; Fig. 4*A*). To quantify this difference, we extracted the functional connectivity of each seed vertex from the FA cluster with each vertex in the DMN and the ExN, bilateral networks defined from elsewhere (*Methods*). Then, we assessed the effect of FA seed hemisphere (left or right), network (DMN or ExN), and network hemisphere (left or right) using a three-factor ANOVA. We found a significant interaction between seed and network factor [*F*(1,455) = 7.77, *P* = 7 × 10^−3^] that confirmed the difference in connectivity in the left and right lOFC with the DMN but not with the ExN observed on the whole-brain maps. Post hoc multiple comparison tests revealed that the right seed was more connected to the DMN compared to the left seed, with no difference in connectivity with the ExN (left versus right seed contrast in relation to DMN, *P* = 1 × 10^−5^; left versus right seed in relation to ExN, *P* = 0.92). The results are depicted in Fig. 4*B*. Additional nonrelevant significant effects are detailed in *SI Appendix*.

In macaques, the same analysis revealed a similar significant interaction between the seed and network factors [seed × network, *F*(1,111) = 14.45, *P* = 2 × 10^−3^]. Post hoc multiple comparison tests revealed that the right seed was more connected to the DMN compared to the left seed, with no difference in connectivity with the ExN (left versus right seed in the DMN, *P* = 2 × 10^−4^; left versus right seed in the ExN, *P* = 0.42). The results are depicted in Fig. 4*D*. Additional nonrelevant significant effects are detailed in *SI Appendix*.

Note that these analyses revealed similar results in both species. However, the underlying pattern of connectivity differs across species. In humans, the left FA cluster is negatively connected to the DMN [left seed with DMN, mean = −0.019, SEM = 0.005, *t* (56) = −3.87, *P* = 3 × 10^−3^; right seed with DMN, mean = 0.001, SEM = 0.004, *t* (56) = 0.37, *P* = 0.71], while in macaques, the left and right FA clusters are positively connected to the DMN [left seed with DMN, mean = 0.062, SEM = 0.008, *t* (13) = 7.93, *P* = 2 × 10^−6^ ; right seed with DMN, mean = 0.11, SEM = 0.017, *t* (13) = 6.57, *P* = 2 × 10^−5^], but the right FA cluster is significantly more positively connected to the DMN.

#### Morphological characteristics in humans.

Given the richness of the HCP data, we were able to further explore some morphological features of the asymmetric FA cluster. We checked whether it was characterized by particular morphological features and found no specific pattern of myelination, gyrification (curvature), or cortical thickness (*SI Appendix*, Fig. S3). We compared such features in the left and right FA cluster and found that the three features of the right FA cluster were higher than in the left FA cluster [myelination, *t* (56) = 3.99, *P* = 2 × 10^−4^; curvature, *t* (56) = 2.49 *P* = 0.016; cortical thickness, *t* (56) = 2.10, *P* = 0.041]. Although there was an asymmetry in those measures, individual variations were not significantly correlated with the RRA, FCA, or FA (mean of RRA and FCA) measures (all *P* > 0.01, threshold for multiple comparisons). Thus, we found no evidence that the morphological differences in the left and right FA clusters are driving the functional asymmetry observed in that particular area.

### Functional Asymmetry and Behavior in Humans.

Here the functional asymmetry of the OFC response to reward has been investigated in relation to the same region’s asymmetrical functional connectivity, in both humans and macaques. The limitations of the HCP reward gambling task used in humans, and notably the simple condition contrast reward versus punishment, restricted our investigation about which specific aspects of reward representation are associated with this lateralization.

We attempted to overcome these limitations by conducting two additional analyses.

First, we aimed at identifying a link between the lOFC asymmetry and specific cognitive process(es) by searching for correlation between 172 behavioral measurements available from the HCP (validated measures that assess cognition, emotion, motor and sensory processes, and personality) and FA coefficients (*Methods*). We did not find any significant effect (all *P* > 0.001, thresholded to account for multiple comparisons). Additionally, this asymmetry was not driven by gender or handedness (gender, *r* = 0.06, *P* = 0.65; handedness, *r* = −0.015, *P* = 0.91).

Then, to identify a putative link between the observed asymmetry and brain functions, we conducted a metaanalysis in Neurosynth by searching for terms associated with the left and right FA clusters (*Methods*). First, we observed that the terms associated with cognitive concepts were different in the left and right lOFC: reward was significantly associated with the left lOFC seed but not with the right lOFC seed [Z(left) = 3.76, Z(right) = 0], and choices had the opposite pattern [Z(left) = 0, Z(right) = 4.88]. Then, we noted that brain regions with a coefficient of metaanalytic coactivation higher than 0.07 with the FA seeds were associated with three types of terms. First, terms showing similar coactivation (food, reward, and olfactory); second, terms associated only with the right lOFC coactivated regions (emotional and affective); and third, terms associated only with the left lOFC coactivated regions (value, taste, monetary, pleasant, and motivational) (*SI Appendix*, Fig. S4). Those observations suggested that both lOFC are involved in reward-related processes and that left lOFC could be more associated to valued-based decision-making and motivational processes, while the right lOFC could be more associated to emotional and affective processes.

## Discussion

In the present study, we provide evidence for functional lateralization in the lOFC in humans and nonhumans primates. Lateralization in the frontal cortex has been considered most often in relation to language processes and praxis (30–32) but also linked to attention (33), cognitive control (34), and emotional regulation (35). In our study, reward responsivity was investigated in the context of decision making. Interestingly, a recent metaanalysis of lateralization of function suggested that decision making rather than emotion, communication, or perception/action is associated with OFC lateralization (16). Although the adaptive consequences of lateralized functions are not well understood, it is thought that hemispheric specialization could increase processing abilities by reducing bilateral redundancy (20).

### RRA and Potential Implications.

RRA in the OFC is consistent with many previous studies investigating the function of the OFC (36–42), yet those studies rarely acknowledge or discuss this observation (43, 44).

Previous studies have suggested that lateralization in the OFC could be linked to valence processing (13), interpreted in an approach/avoidance framework (44) or in an exploration/exploitation framework (39). Yet our Neurosynth metaanalysis suggested that value-guided and motivational processes might be more supported by the left lOFC, while the right lOFC has more of a role in emotional and affective processes. It should nevertheless be reminded that some studies have reported no effect of OFC lesion laterality (45) or bilateral OFC responses to reward (46, 47). Therefore, we do not claim an absolute and total functional dissociation between left and right lOFC but rather a graded difference between the contributions that they make. It is possible that the relative contribution of each hemisphere’s lOFC might differ depending on the requirements of the experimental paradigm. This last point is supported by the protocol-specific hemispheric dominance observed in macaques. Each individual macaque exhibits a degree of functional lateralization in the lOFC region, but the direction in which it manifests varies across task protocols. We found a rightward asymmetry in monkeys performing a probabilistic two-choices learning task versus a left asymmetry in monkeys doing a value-based forced choice experiment. Thus, the task at stake could differentially recruit the left and right lOFC. For instance, some studies only report the left OFC to represent outcome information (48), while others only report the right OFC to respond to identity-specific value (41, 42). However, because the protocols and contrasts that we used vary on several factors, while we are certain that hemispheric difference exists, future studies will need to specifically investigate its precise basis.

Alternatively, the RRA difference between species could point to distinct lateralization of brain circuits in macaques compared to humans. Although recent evidence suggests that behavioral and structural or functional asymmetries also exist in nonhuman species (23, 49, 50), asymmetries specific to some species could also be observed, for instance, for spatial processing in macaques (25) or regarding the architecture supporting language processing in humans (51).

Crucially, we show an interrelationship across subjects between the RRA and a functional connectivity-related asymmetry (FCA). This result—a relationship between two very different indices of lateralization—suggests that the lateralization observed in the context of reward processing is likely to be also observed in other conditions requiring the involvement of the lOFC.

### FCA and Potential Implications.

Given that connectivity constrains and partly determines the functions that could be supported by a given brain region (52), we used rs-MRI results to further speculate about the nature of the functional differences between the left and right lOFC.

Differences between connectivity patterns in the left and right lOFC are notably related to their coupling with a set of brain regions often referred to as the DMN. The right lOFC was found to be more strongly connected to the DMN than the left lOFC.

The DMN has been shown to strongly overlap with the social brain network (53). Therefore, the differential connectivity with the DMN might suggest a differential contribution of the left/right lOFC to social cognition. Some lesion studies in humans support this framework. For instance, patients with right-sided orbitofrontal lesions had more profound disturbances of social and interpersonal behavior than patients with a left-sided lesion (14). However, in fMRI studies, responses to social feedbacks, if anything, appear stronger in the left OFC than in the right OFC (54). Moreover, neither previous metaanalysis (55) nor the presented Neurosynth metaanalysis support this framework.

DMN has also been associated with self-referential mental activity and recollection of prior experiences (56). It might therefore be hypothesized that it is an internally versus externally driven valuation process that underlies the lOFC functional asymmetry: value assignment that requires individuals to remember or simulate (such as the taste of a cake) could recruit more the right lOFC. On the other hand, a valuation process linked to external features, such as color combination in a painting, could recruit more the left lOFC. Future investigations will aim at testing this specific hypothesis.

In humans, we observed a stronger negative functional connectivity with the DMN in the left lOFC compared to the right lOFC. Brain regions showing negative functional connectivity with the DMN usually belong to networks activated for tasks that demand mental control or attention (57). Interestingly, we did not find significant difference between the left and right lOFC regarding their functional connectivity with the ExN. This suggests that the left lOFC might correspond to a functional hub at the interface between the DMN and the ExN.

Finally, another potential functional distinction between the left and right lOFC comes from its links with the control of autonomic responses. Tracing studies in nonhuman primates have linked the medial more strongly than the lateral part of the OMPFC to the autonomic nervous system (58–60). Nevertheless, a study focusing on mediolateral differences in OFC–hypothalamus connectivity suggested the possibility of some differences in the lOFC region we focus on here (61).

## Conclusion

In summary, OFC lateralization has been overlooked or mentioned only in passing in many functional studies. Here we provide evidence for lateralization in terms of reward-related function and in terms of functional connectivity both in humans and in macaques. The observation of this result in both species suggests that this asymmetry could have been present in the last common ancestor of humans and old-world monkeys around 29 Mya. A recent study found an interhemispheric OFC asymmetry in rodents in a reversal learning task (62), with the right OFC being more recruited in the task than the left OFC. In tandem with our current results, this suggests that RRA in or near lOFC might have been a feature of the mammalian brain present since the last common ancestor of rodents and primates more than 100 Mya. Altogether, we strongly encourage future studies to report relative variation in activation in the left and right OFC and to consider differences between hemispheres when interpreting results in OFC.

## Methods

### Subjects.

#### Humans.

The data used in this study are released as part of the Washington University - University of Minnesota Consortium of the Human Connectome Project (WU-Minn HCP, RRID SCR_008749, https://www.humanconnectome.org/study/hcp-young-adult) (51). We selected the S900 subject release with 7T structural and rs-MRI data. The data were preprocessed according to the HCP pipeline (52). Of the 73 subjects in this specific HCP release, 16 subjects were excluded because of family ties with other subjects in the database. The data analysis was therefore based on 57 subjects (37 females).

Analyses were conducted on the data aligned using areal feature-based registration [called multimodal surface matching (MSMAll) (29)]. This procedure aligns vertices on the cortical surface across subjects not only according to gross folding morphology but also taking into account the subject-specific functional features, such as the location and distribution of resting-state networks. The MSMAll approach dramatically improves the functional alignment of cortical areas over and above registration based solely on volumetric or surface-based morphological registration. This type of registration is referred to as area-based registration and is sometimes considered a near-optimal functional alignment (29).

#### Macaques.

Fourteen rhesus monkeys (*M. mulatta*, 13 males) were involved in the study. They weighed 7 to 14 kg and were 7 to 13 y of age. They were group housed and kept on a 12-h light–dark cycle, with access to water 12 to 16 h on testing days and with no restriction of access on nontesting days. All procedures were conducted under licenses from the UK Home Office in accordance with the UK The Animals (Scientific Procedures) Act 1986 and with European Union guidelines (EU Directive 2010/63/EU). Among the 14 monkeys, 8 participated in four different experimental tasks (protocols). The detail of assignment of monkeys to the different tasks is described in Table 1.

**Table 1.** Monkey ID and protocol details

Monkey ID	rs-MRI	fMRI	Protocol ID	Number of sessions	Number of contrasts of interest
1	1	0	—	—	—
2	1	0	—	—	—
3	1	1	4	12	2
4	1	1	1	10	1
5	1	1	1	10	1
6	1	1	1	10	1
7	1	1	1	10	1
8	1	0	—	—	—
9	1	0	—	—	—
10	1	1	4	13	2
11	1	0	—	—	—
12	1	0	—	—	—
13	1	1	2	12	2
			3	11	2
14	1	1	2	11	2
			3	10	2
			4	11	2
Total	14	8		200	18

### Experimental Tasks.

#### Gambling task in humans.

Reward-related BOLD (blood-oxygen-level dependent) signal was recorded with fMRI during a card-guessing gambling task played for monetary reward that has been previously described (26). Participants completed a card-guessing game where they were required to guess the number (ranging from 1 to 9) on a mystery card in order to win or lose money. Participants were instructed to guess if the unknown card number was more or less than 5 by pressing one of two buttons on a response box with their right hand. Feedback was given as the revealed card number with a cue to inform the participants if they received a monetary reward, monetary loss, or nothing (neutral no reward/loss outcome received for number 5) trial. The task was presented in blocks of eight trials that were either mostly rewarded (six reward trials pseudorandomly interleaved with neutral and/or loss trials) or mostly loss (six loss trials interleaved with reward and/or loss trials). For each of the two runs, there were two mostly reward and two mostly loss blocks, interleaved with four fixation blocks (15-s duration).

#### Protocol 1 in monkeys: Object discrimination reversal task.

The experimental task used in protocol 1 is described in detail elsewhere (40, 63). Briefly, the task was designed to investigate contingent learning mechanisms and specifically how and where in the brain associations between choice options and outcomes (i.e., reception of reward) resulting from choosing them are formed. Four macaques had to choose between pairs of abstract visual stimuli while in the MRI scanner. On each trial, the two stimuli available for choice (available options) were drawn from a set of three, each associated with distinct reward probabilities. The rewards were delivered probabilistically in a manner that fluctuated across the session, with two of the options reversing toward the middle of a session. Each stimulus’s reward probability was uncorrelated from that of the others. On each trial one of the two available options was chosen by the monkey, the other was unchosen, and a third option was invisible and unavailable for choice. In our study, we focused on the receipt of the reward.

#### Protocols 2 and 3 in monkeys: Decision to act task.

The experimental task used in protocol 2 is described in detail elsewhere (64). Briefly, the task was designed to investigate how contextual factors and internal state, shaped by present and past environment, are integrated to influence whether and when to act. Four monkeys initially performed this task, but we only included the two monkeys (13, 14) who also performed the resting-state fMRI data acquisition. In that task, macaques were trained to track the number of dots on a screen while in the MRI scanner. Dots appeared one at a time on a screen, and animals could decide to make a response, at a time of their choice, by tapping on a response pad in front of them. The number of dots on the screen at the time of response determined the probability of reward. Reward probability was drawn from a sigmoid function: the longer the animals waited before responding, more dots appeared on the screen, and the higher was the probability of reward. Different levels of reward magnitude were associated with different dot colors, and the reward magnitude varied from trial to trial. Once the monkeys responded, they received drops of juice or no juice according to the reward probability distribution and the time of their response. There was a 4-s delay between the response and the outcome. In the context of our study, two events on each trial were of special interest: the onset of the stimulus (dots), since the color is indicating the expected level of reward, and the outcome (zero, one, two, or three drops of blackcurrant juice).

Data from protocol 3 have not been published yet, but data supporting our results have been deposited in a publicly accessible database (https://osf.io/ybm4s). However, the task is exactly the same except that the frequency of all of the good offers increased, and that of all of the bad offers decreased (i.e., there were more trials with high reward magnitude and fewer trials with low reward magnitude in protocol 3 compared to protocol 2).

#### Protocol 4: Stimulus–reward association task.

The data and results from the experimental task used in protocol 4 have not been published yet, but data supporting our results have been deposited in a publicly accessible database (https://osf.io/ybm4s). Briefly, the control task used here investigated how a monkey would respond to visual cues indicative of how much reward could be obtained or lost (i.e., poured into a visible plastic jar). Four male rhesus macaques were trained to associate a set of 10 stimuli with various reward magnitudes (i.e., from zero to two drops of reward smoothie that could be either obtained or discarded). On any trial, one stimulus was presented on the screen. The monkey had 10 s to respond by putting his hand over a homemade infrared sensor. Once selected, the stimulus was replaced by a hollow white frame. After a 3.5- to 4.5-s delay, the stimulus was presented back (feedback) and the reward delivered. If the monkey did not respond within 10 s, the trial was aborted, and the same stimulus was presented again after the intertrial interval. The stimulus–outcome association was probabilistic. In 24% of the trials, the feedback was different from the cue. The obtained reward was always congruent with the displayed feedback.

In all macaque protocols, monkeys were responding by making hand movements over a homemade infrared sensitive sensor. They were free to use both hands to respond.

#### fMRI data acquisition, processing, and analysis in humans.

The preprocessed 3T data were downloaded from the HCP website for the 57 selected subjects. For each subject, the fMRI data were preprocessed using the HCP functional pipeline, including the volume and MSMAll surface pipeline outputs, motion parameters, and FMRIB’s Software Library (FSL; RRID SCR_002823) (65) files for higher analysis. All preprocessing steps and preliminary analysis are described in ref. 26. Briefly, the HCP fMRIVolume pipeline performs gradient unwarping, motion correction, field map unwarping, and grand mean intensity normalization on the four-dimensional time series. These volumes are segmented (Brain Boundary Registration), registered to the T1 anatomical volume using nonlinear image registration tool (FNIRT), and warped to standard (MNI152) space. Parameter estimates were estimated for a preprocessed time series using a general linear model (GLM) using FMRIB’s improved linear model with autocorrelation correction. Predictors were convolved with a double gamma canonical hemodynamic response function to generate regressors. Temporal derivatives of each regressor were added to the GLM as covariates of no interest. Parameter estimates (BOLD) for the contrast (reward > punishment; cope6.feat) were available for 57 participants. As the task design consisted in blocks of eight trials that were either mostly associated with reward (75% of rewarded trials) or mostly associated with a loss (75% of losing trials), the blocks were labeled “gain” and “loss,” respectively. Contrasting activity from these two blocks identifies a general difference between positive outcomes (gains) versus negative outcomes (losses). We chose this contrast to establish relationships with reward, in accordance with the initial purpose of the experimental paradigm used in the HCP. In addition, we checked the opposite contrast to identify regions that would be more specifically sensitive to punishment.

To obtain group statistics, second-level (group) analysis on volumes was conducted using FMRIB’s Local Analysis of Mixed Effects stage 1, part of FSL (version 5.0.8, https://fsl.fmrib.ox.ac.uk/fsl/fslwiki/). The main contrast of interest, reward versus punishment, of each participant was entered into a second-level random-effects analysis using a one-sample *t* test. Z statistic images were thresholded using clusters determined by z > 2.3 and a corrected cluster significance threshold of *P* = 0.05.

For clarity in the data visualization and for a better visual comparison with resting-state data, we then projected the volume result on the averaged MSMAll midthickness surface of all participants, using the “wb command” and “volume to surface mapping” functions from the Connectome Workbench (https://www.humanconnectome.org/software/connectome-workbench).

To test the asymmetry of reward-related activity, each individual z-stat map corresponding to the reward versus punishment contrast was projected onto its corresponding MSMAll surface. Then, the left and right data were extracted from each hemisphere in the OMPFC. The individual unsigned differences between the left and right z statistics in the OMPFC were computed and then assessed for significance at the group level using permutation tests (see *Statistical assessment*).

#### fMRI data acquisition and processing in macaques.

Awake animals were head-fixed in a sphinx position in an MRI-compatible chair (Rogue Research). MRI was collected using a 3T horizontal bore MRI clinical scanner and a four-channel phased array receive coil in conjunction with a radial transmission coil (Windmiller Kolster Scientific). Each loop of the coil had an 8-cm diameter, which ensures a good coverage of the animal’s head. Similar coils have been previously used for awake fMRI studies in primates (40, 66, 67). The chair was positioned on the sliding bed of the scanner. The receiver coils were placed on the side of the animal’s head with the transmitter placed on top. An MRI-compatible screen (MRC) was placed 30 cm in front of the animal, and the image was projected on the screen by a LX400 projector (Christie Digital Systems). Functional data were acquired using a gradient-echo T2* echo planar imaging (EPI) sequence with a 1.5 × 1.5 × 1.5 mm resolution, repetition time (TR) 2.28 s, echo time (TE) 30 ms, and flip angle 90°. At the end of each session, proton density-weighted images were acquired using a gradient-refocused echo (GRE) sequence with a 1.5 × 1.5 × 1.5 mm resolution, TR 10 ms, TE 2.52 ms, and flip angle 25°. These images were later used for offline MRI reconstruction.

Preprocessing was performed using tools from FSL (68), Advanced Normalization Tools (http://stnava.github.io/ANTs) (69), HCP Workbench (70) (https://www.humanconnectome.org/software/connectome-workbench), and the Magnetic Resonance Comparative Anatomy Toolbox (https://github.com/neuroecology/MrCat). First, T2* EPI images acquired during task performance were reconstructed by an offline sensitivity encoding (SENSE) method that achieved higher signal-to-noise and lower ghost levels than conventional online reconstruction (71) (Offline_SENSE MatLab App, Windmiller Kolster Scientific). A low-noise EPI reference image was created for each session, to which all volumes were nonlinearly registered on a slice-by-slice basis along the phase-encoding direction to correct for time-varying distortions in the main magnetic field due to body and limb motion. The aligned and distortion-corrected functional images were then nonlinearly registered to each animal’s high-resolution structural images. A group-specific template was constructed by registering each animal’s structural image to the CARET macaque F99 space (71). Finally, the functional images were temporally filtered (high-pass temporal filtering, 3-dB cutoff of 100 s) and spatially smoothed (Gaussian spatial smoothing, full-width half-maximum of 3 mm).

#### fMRI data analysis in macaques.

To perform whole-brain statistical analyses we used a univariate GLM framework as implemented in FSL FMRI Expert Analysis Tool (FEAT) (72). At the first level, we constructed a GLM to compute the parameter estimates for each regressor. The GLMs were constructed based on the specific questions raised in each protocol and are detailed in *SI Appendix*, Table S1. From protocol 1, we used the contrast reward versus no reward, time-locked to the onset of feedback. For protocols 2, 3, and 4, we used two contrasts linked to parametric modulators: expected reward level, time-locked to the stimulus onset (or decision onset for protocol 4), and received reward level, time-locked to reward delivery.

For each protocol and each contrast, the first-level z statistics of each session in every monkey were extracted to compute the main effect of reward (mixed-effect analysis). Then, each z-statistic volume was projected onto left and right surfaces and used to compute the asymmetry of reward representation in the OMPFC (linear mixed-effect models that include random factor for protocol and monkeys).

#### rs-MRI data acquisition and processing in humans.

The preprocessed 7T data were downloaded from the HCP website. We selected the package called “Resting State fMRI 1.6 mm/59k FIX-Denoised (compact),” which contained 1.6-mm resolution data. The rs-fMRI acquisitions (including the use of leading-edge, customized MRI hardware and acquisition software) and image processing are covered in detail in refs. 70, 73, 74. After image preprocessing [primarily using FSL (RRID SCR_002823) (68), FreeSurfer (RRID SCR_001847) (75), and Connectome Workbench (76) software packages], the functional time series are filtered, and artifacts are removed using an automated data-driven approach that relies on Independent Component Analysis (ICA) decomposition and hand-trained hierarchical classification (FMRIB’s ICA-based X-noisifier [FIX]) (73). We concatenated the MSMAll data from the four available resting-state sessions (de-meaned then concatenated) to obtain one time series per participant.

#### rs-MRI data acquisition and processing in macaques.

The 14 monkeys were scanned under anesthesia to acquire resting-state data. fMRI and anatomical scans were collected according to previously used protocols (77). Anesthesia was induced using intramuscular injection of ketamine (10 mg/kg) combined with either xylazine (0.125 to 0.25 mg/kg) or midazolam (0.1 mg/kg) and buprenorphine (0.01 mg/kg). Macaques also received injections of atropine (0.05 mg/kg), meloxicam (0.2 mg/kg), and ranitidine (0.05 mg/kg). Anesthesia was maintained with isoflurane. Isoflurane was selected because it has been demonstrated that resting-state networks are still present using this agent for anesthesia (78). The anesthetized animals were placed in an MRI-compatible stereotactic frame (Crist Instrument) in a sphinx position within a horizontal 3T MRI scanner with a full-size bore. The same coils as for awake scans (*fMRI data acquisition and processing in macaques*) were used for data acquisition. Whole-brain BOLD fMRI data were collected using the following parameters: 36 axial slices, resolution of 1.5 × 1.5 mm, slice thickness of 1.5 mm, TR of 2,280 ms, TE of 30 ms, 1,600 volumes. Structural scans were acquired in the same session using a T1-weighted MP-rage sequence (no slice gap, 0.5 × 0.5 × 0.5 mm, TR of 2,500 ms, TE of 4.01 ms, and 128 slices).

The detailed preprocessing pipeline for the resting-state fMRI has been described elsewhere (79, 80). Briefly, after reorientation to the same convention for all functional EPI datasets, the first volumes were discarded to ensure a steady radio frequency excitation state. EPI time series were motion corrected using Motion Correction in FMRIB's Linear Image Registration Tool (MCFLIRT) (81). Brain extraction, bias correction, and registration were achieved for the functional EPI datasets in an iterative manner, and the mean of each functional dataset was registered to its corresponding T1w image using rigid-body boundary-based registration [FMRIB's Linear Image Registration Tool, FLIRT (81, 82)]. EPI signal noise was reduced in both the frequency and temporal domain. The functional time series were high-pass filtered with a frequency cutoff at 2,000 s. Temporally cyclical noise, for example, originating from the respiration apparatus, was removed using band-stop filters set dynamically to noise peaks in the frequency domain of the first three principal components of the time series. To account for remaining global signal confounds we considered the signal time series in white matter and meningeal compartments; there confound parameters were regressed out of the BOLD signal for each voxel. Following this confound cleaning step, the time series were low-pass filtered with a cutoff at 10 s. The data were transformed to F99 and spatially smoothed using a 2-mm full-width at half-maximum Gaussian kernel. Last, the data time series were de-meaned to prepare for functional connectivity analyses.

#### rs-MRI data analysis.

All analyses and statistics were conducted in MATLAB 2018b (MATLAB and Statistics Toolbox Release 2017a, The MathWorks, Inc., RRID SCR_001622, https://www.mathworks.com/) with in-house bespoke scripts calling Workbench executables.

Group analyses using MELODIC’s (Multivariate Exploratory Linear Optimized Decomposition into Independent Components) incremental group-principal component analysis (PCA) (MIGP) were first conducted to investigate the global patterns of asymmetry in the OMPFC. MIGP analysis corresponds to a group PCA, as described in ref. 83. The brain activity time series of each participant are sequentially included in a PCA analysis in order to provide a close approximation to the full concatenation of all participant time series, without large memory requirements. The output of this analysis is a time series of similar size to an individual time series.

#### Network definition.

In humans, to assess the connectivity of regions of interest to the DMN and to the ExN, the names of the two networks were entered as a topic term in https://www.neurosynth.org/, and the association (for the DMN) and uniformity test (for the ExN) maps were downloaded. Maps were then projected onto surfaces and thresholded for clusters bigger than 100 vertices.

In macaques, the networks were defined from the connectivity of bilateral seeds in the anterior cingulate sulcus (DMN) and the midcingulate sulcus (ExN).

#### Region of interest definition.

Regions of interest (ROIs) were drawn manually on the vmPFC and the OFC, to cover a large portion of the OMPFC. The dorsal medial boundary was delineated by an arbitrary horizontal line that runs from the front of the brain to the genu of the corpus callosum. The ventral surface of the frontal lobe was included from the frontal pole rostrally to the anterior perforated substance caudally.

#### FCA coefficient.

To investigate the asymmetry of connectivity between the left and the right OMPFC, the FCA coefficient was computed as follows:

$${\text{FCA}}_{Ipsi}=\frac{\sum_{j=1}^{m}\left|C_{OL}^{WB}\left(j\right)-C_{OR}^{WB}\left(j\right)\right|}{m},$$

with m the number of vertices in the whole brain (WB) and $C_{OX}^{WB}$(j) the connectivity of every n vertices of the X (left or right) OMPFC with a vertex j of the brain. FCA is a vector of n elements, graphically represented on the heat maps in Fig. 2.

The stability of the measure was assessed by comparing four additional ways of computing FCA. All measures converged toward the same result (*SI Appendix*, Fig. S5).

#### Statistical assessment.

The statistical validity of our results was assessed by extracting variables of interest from each subject and testing for significance at the group level using one-sample *t* tests. When assessing significance of clusters on rs-MRI data, the FCA map was computed for every subject. The main effect was then tested using one-sample Student *t* test (two-tailed).

To assess the statistical validity of the RRA clusters in both humans and monkeys, we used the Fisher randomization test (84) with 10,000 randomizations of the RRA values (z-scored) of each subject. The maximal cluster-level statistics (the sum of t values across contiguous points passing a significance threshold of 0.01 [z = 2.3]) were extracted for each shuffle to compute a null distribution of effect size across the OMPFC mask. For each significant cluster in the original (nonshuffled) data, we computed the proportion of clusters with higher statistics in the null distribution, which is reported as the cluster-corrected *P* value (p_corr_) (85).

#### Anatomical MRI data acquisition and analyses.

The preprocessed anatomical 7T data were downloaded from the HCP website. We selected the package called “Structural Preprocessed for 7T (1.6 mm/59k mesh),” which contained 1.6-mm resolution data, collected at 3T. In this package, myelin, curvature, and cortical thickness maps are available for each subject, registered on MSMAll, making those maps comparable with the connectivity maps. When investigating the morphological features of the OMPFC, we extracted the values of those maps for each subject and computed the mean of each feature.

#### Control analyses.

In both human and macaque fMRI and rs-MRI data, we checked for hemispherical differences in the signal-to-noise ratio (SNR) in the FA cluster. None of the difference in SNR was significant (all *P* > 0.05; *SI Appendix*, Fig. S6).

#### Behavioral analyses.

Open access behavioral data were downloaded from the HCP website. Pearson correlation coefficients were computed between the 172 available behavioral measures and the individual FA coefficients (mean of RRA and FCA coefficients). To account for multiple comparisons, we set the significance threshold at *P* = 0.001.

We also checked in humans that there was no link between lateralization and handedness or gender.

#### Neurosynth metaanalysis.

We extracted the MNI coordinates of the FA cluster and mirrored it to obtain one ROI for each hemisphere (left, x = −28, y = 44, z = −12; right, x = 28, y = 44, z = −12). We entered those coordinates into Neurosynth (https://neurosynth.org/) as locations and extracted two individual seed-based variables linked to terms for each of them: the significant z scores (associated to the seed) and the metaanalytic coactivation coefficients higher than 0.07 (regions coactivated with the seed). This resulted in a list of terms for each seed. We removed the terms corresponding to the name of brain structures (such as “OFC” or “orbitofrontal”) and then looked for the values of terms present in both seeds and absent in one ROI but present in the other one.

This procedure resulted in 2 terms for the z scores (reward and choices) and 10 terms for the metaanalytic coactivation coefficients (food, reward, value, taste, olfactory, monetary, pleasant, motivational, emotional, and affective).

## Supplementary Material

Supplementary File

## Data Availability

All human data are publicly available from https://www.humanconnectome.org/study/hcp-young-adult.. Scripts and data (human and macaque) supporting the results of our study are available at Open Science Framework (https://osf.io/ybm4s) (86). Human meta-analytical maps are available at Neurosynth (https://neurosynth.org/locations/28_44_-12_6 and https://neurosynth.org/locations/-28_44_-12_6).
