# Supplementary material for: Differential functional connectivity underlying asymmetric reward-related activity in human and nonhuman primates
Source: Proc Natl Acad Sci U S A. 2020 Oct 29;117(45):28452–62. doi: 10.1073/pnas.2000759117 (PMC7668182; doi:10.1073/pnas.2000759117)
Supplement: Supplementary File [file pnas.2000759117.sapp.pdf]

PNAS
www.pnas.org

Supplementary Information for

Differential functional connectivity underlying asymmetric reward-related activity in human and non-human primates

Alizée Lopez-Persem[1,2]*, Léa Roumazeilles[1], Davide Folloni[1], Kévin Marche[1], Elsa F. Fouragnan[3], Nima Khalighinejad[1], Matthew F. S. Rushworth[1], Jérôme Sallet[1,4]*

[1] Wellcome Integrative Neuroimaging Centre, Department of Experimental Psychology, University of Oxford, Oxford, UK
[2]FRONTLAB, Institut du Cerveau (ICM), Sorbonne Université, INSERM, CNRS, Hôpital Pitié Salpêtrière, Paris, France
[3] School of Psychology, University of Plymouth, Plymouth, UK
[4] Univ Lyon, Université Lyon 1, Inserm, Stem Cell and Brain Research Institute U1208, Bron, France

* Corresponding authors: lopez.alizee@gmail.com and jerome.sallet@psy.ox.ac.uk

This PDF file includes:

Supplementary text
Figures S1 to S6
Table S1

Supplementary Information Text

Hemispheric dominance in the lOFC functional connectivity

For both species, we computed a signed difference of functional connectivity of the left and right lOFC (absolute functional connectivity of the right lOFC minus absolute functional connectivity of the left lOFC). We extracted this signed FCA measure for each individual. This analysis revealed no significant direction of asymmetry in humans (Right-Left mean=0.001, SEM=0.0036, t(56)=0.583, p=0.57) but a significant right dominance in macaques (Right-Left mean=0.0135, SEM=0.0041, t(13)=3.26, p=0.006). This result is consistent with the maps displayed in Figure 4 A and C, in which absolute functional connectivity strengths (yellow and green) are more pronounced in the brain map representing the functional connectivity of the right lOFC in macaques but not in humans. This analysis reveals that in macaques, the right lOFC is more connected to the rest of the brain than the left lOFC.

Functional connectivity asymmetry stability assessment.

To investigate the stability of the asymmetry of connectivity between the left and the right OMPFC, four measures of asymmetry were used:

- Ipsilateral Functional Connectivity Asymmetry (FCA_{Ipsi}): Difference between the connectivity of the left OMPFC (OL) with the left hemisphere (HL) and the right OMPFC (OR) with the right hemisphere (HR).

$$FCA_{Ipsi} = \frac{\sum_{j=1}^{m} |C_{OL}^{HL}(j) - C_{OR}^{HR}(j)|}{m}$$

- Contralateral Functional Connectivity Asymmetry (FCA_{contra}): Difference between the connectivity of the left OMPFC (OL) with the right hemisphere (HR) and the right OMPFC (OR) with the left hemisphere (HL).

$$FCA_{Contra} = \frac{\sum_{j=1}^{m} |C_{OL}^{HR}(j) - C_{OR}^{HL}(j)|}{m}$$

- Left Functional Connectivity Asymmetry (FCA_{Left}): Difference between the connectivity of the left OMPFC (OL) with the left hemisphere (HL) and the right OMPFC (OR) with the left hemisphere (HL).

$$FCA_{Left} = \frac{\sum_{j=1}^{m} |C_{OL}^{HL}(j) - C_{OR}^{HL}(j)|}{m}$$

- Right Functional Connectivity Asymmetry (FCA_{Right}): Difference between the connectivity of the left OMPFC (OL) with the right hemisphere (HR) and the right OMPFC (OR) with the right hemisphere (HR).

We found in each of the four resulting maps of human OMPFC functional connectivity at least one cluster in the OFC with a particularly high asymmetry. The conjunction of the four maps revealed a unique cluster (Figure S4). In the following analyses, the FCA measure corresponds to the average of the four types of asymmetry measures. We confirmed the significance of FCA in this cluster at the group level in humans (t(56)=12.29, p=2.10^{-17}). The same analysis conducted in macaque data revealed very similar results; the conjunction analysis showed a single cluster in the OFC, with a significant FCA at the group level (t(13)=3.01, p=0.01).

Functional connectivity pattern of the left and right FA clusters

The 3-factors ANOVA conducted on the connectivity profiles of the left and right FA clusters revealed the following main effects and interactions:

- In humans:
    - Seed hemisphere : $F(1,455)=14.41$, $p=0.0004$
    - Network: $F(1,455)=132.33$, $p=2.10^{-16}$
    - Network hemisphere: $F(1,455)= 53.36$, $p=1.10^{-9}$
    - Seed hemisphere * Network: $F(1,455)= 7.77$, $p=0.0072$
    - Seed hemisphere * Network hemisphere : $F(1,455)= 7.79$, $p=0.0072$
    - Network * Network hemisphere : $F(1,455)= 29.19$, $p=1.10^{-6}$
    - Seed hemisphere * Network * Network hemisphere: $F(1,455)= 3.48$, $p=0.0675$

- In macaques:
    - Seed hemisphere: $F(1,111)=4.77$, $p=0.0478$
    - Network: $F(1,111)=25.95$, $p=0.0002$
    - Network hemisphere: $F(1,111)=40.19$, $p=2.10^{-5}$
    - Seed hemisphere * Network: $F(1,111)=14.45$, $p=0.0022$
    - Seed hemisphere * Network hemisphere: $F(1,111)=1.87$, $p=0.1942$
    - Network * Network hemisphere: $F(1,111)=25.75$, $p=0.0002$
    - Seed hemisphere * Network * Network hemisphere: $F(1,111)=0.36$, $p=0.5587$

Signal-to-Noise ratio (SNR) estimation

In humans, the SNR was estimated as follow:

- fMRI:

$$tSNR_{fMRI} = \frac{\sum_{i=1}^{s} \frac{mean(TS(wb,i))}{std(TS(wb,i))}}{s}$$

- rs-MRI

$$tSNR_{rsMRI} = \frac{\sum_{i=1}^{s} \frac{mean(TS(wb,i))}{std(TS(wb,i))}}{s}$$

With s the number of sessions in each scanning procedure (four), TS(wb,i) the timeseries recorded in each session for all vertices (wb: whole brain).

In macaques, as the preprocessing steps removed the mean from the time series, we estimated the connectional SNR for the rs-MRI data as follow:

$$\mathrm{CM} = \frac{\sum_{c=1}^{t} \frac{\sum_{j=1}^{m} |TS(j,c) * TS(wb,c)|}{m}}{t}$$

$$cSNR = \frac{mean(CM)}{std(CM)}$$

With CM the average connectional map across 12 partitions (c) of the time series TS of each vertex j. wb: whole brain. cSNR: connectional SNR.
As the scanning parameters were the same in rs-MRI and fMRI sessions across macaques, we did not estimate the cSNR for macaque fMRI data.

The SNR maps obtained from those measures are displayed in Figure S5. We then computed the hemispheric difference between SNR and found no significant difference between the left and right FA clusters (human SNR_{fMRI}: $t(72)=1.93$, $p=0.06$, human SNR_{rsMRI}: $t(56)=0.36$, $p=0.72$, macaque cSNR: $t(13)=-1.54$, $p=0.15$).

Fig. S1.

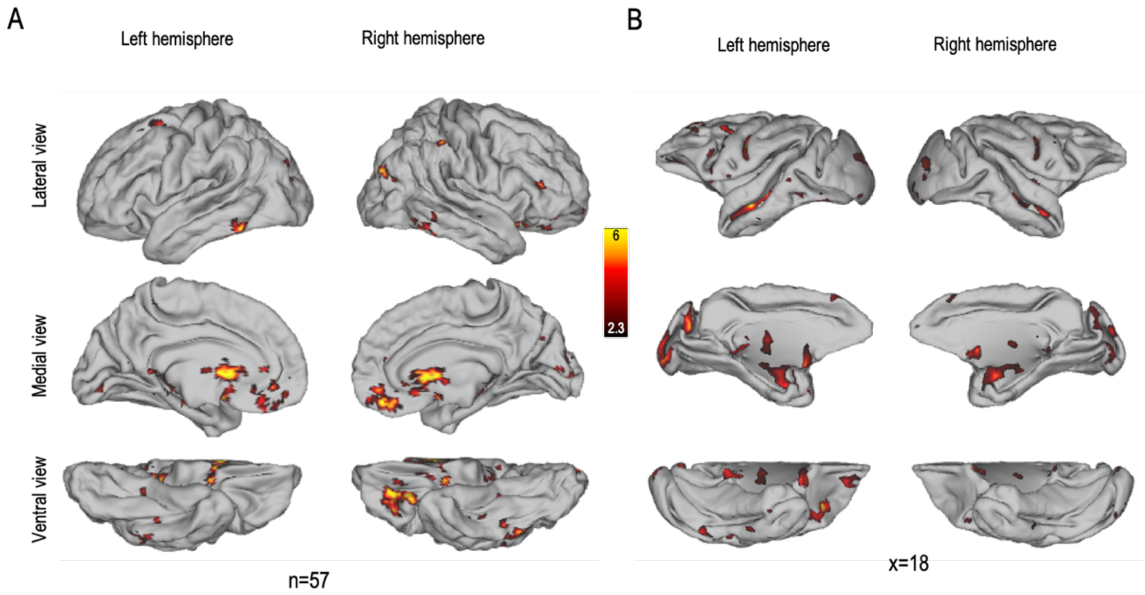

Figure S1 - Neural responses to reward in humans and macaques.
A. Statistical maps showing positive effect of the contrast 'reward versus punishment' in humans ($z>2.3$, cluster-corrected). n indicates the number of subjects. B. Statistical maps showing positive effect of the reward regressors in macaques ($z>2.3$, cluster-corrected). x indicates the number of experimental points across protocols (see Methods).

Fig. S2.

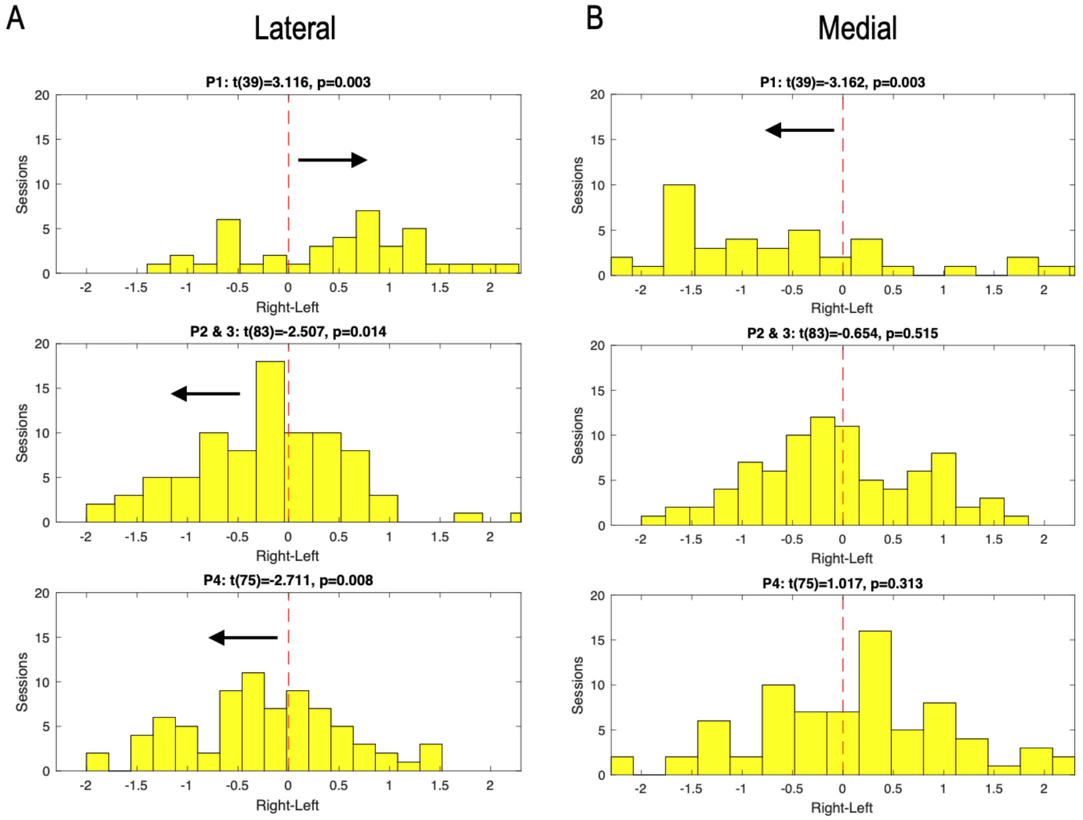

Figure S2. Direction of reward-related asymmetry type of protocols in the lateral and medial OFC cluster. A. Histograms of individual session signed RRA coefficients grouped per protocol (P1 to P4) in the lateral OFC cluster. B. Same but for the medial OFC cluster. Arrows indicate the direction of a significant signed RRA.

Fig. S3.

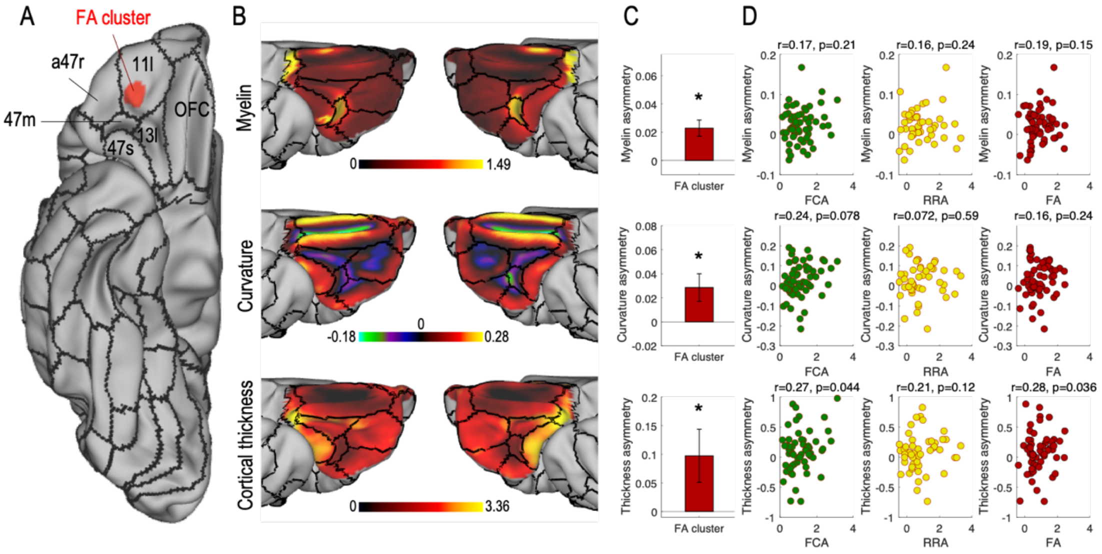

Figure S3 – Morphological characteristics of the human FA cluster.
A. Overlap of the FA cluster (red) and the parcellation from Glasser et al 2016 (29) (black borders). **B**. Morphological features of the OMPFC: Myelin, Curvature (negative in sulci, positive on gyri) and cortical thickness. **C**. Signed difference between left and right morphological features. Star indicates significance against 0. Bar represent the mean across subjects and error bars represent SEM across subjects. **D**. Morphological asymmetries in function of FCA (green), RRA (yellow), and the average of the two measures (FA, red) in the FA cluster.

Fig. S4.

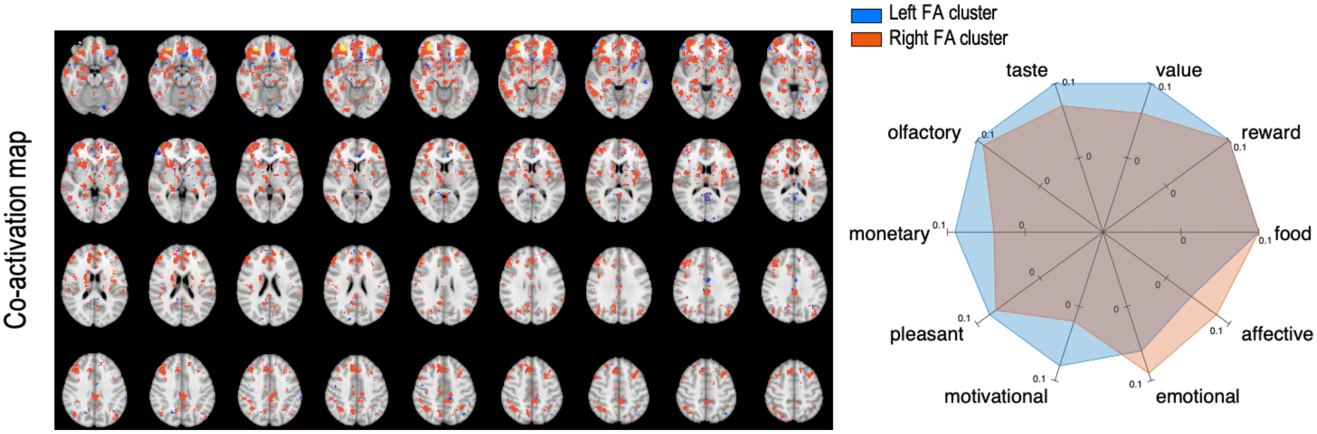

Figure S4. Meta-analytic co-activation maps and terms associated to the left and right FA clusters.

Left. Neurosynth meta-analytic co-activation maps associated to the left (blue) and right (orange) FA clusters. Right. Spider-plot representing the meta-analytic co-activation coefficients for all terms associated to the FA clusters.

Fig. S5

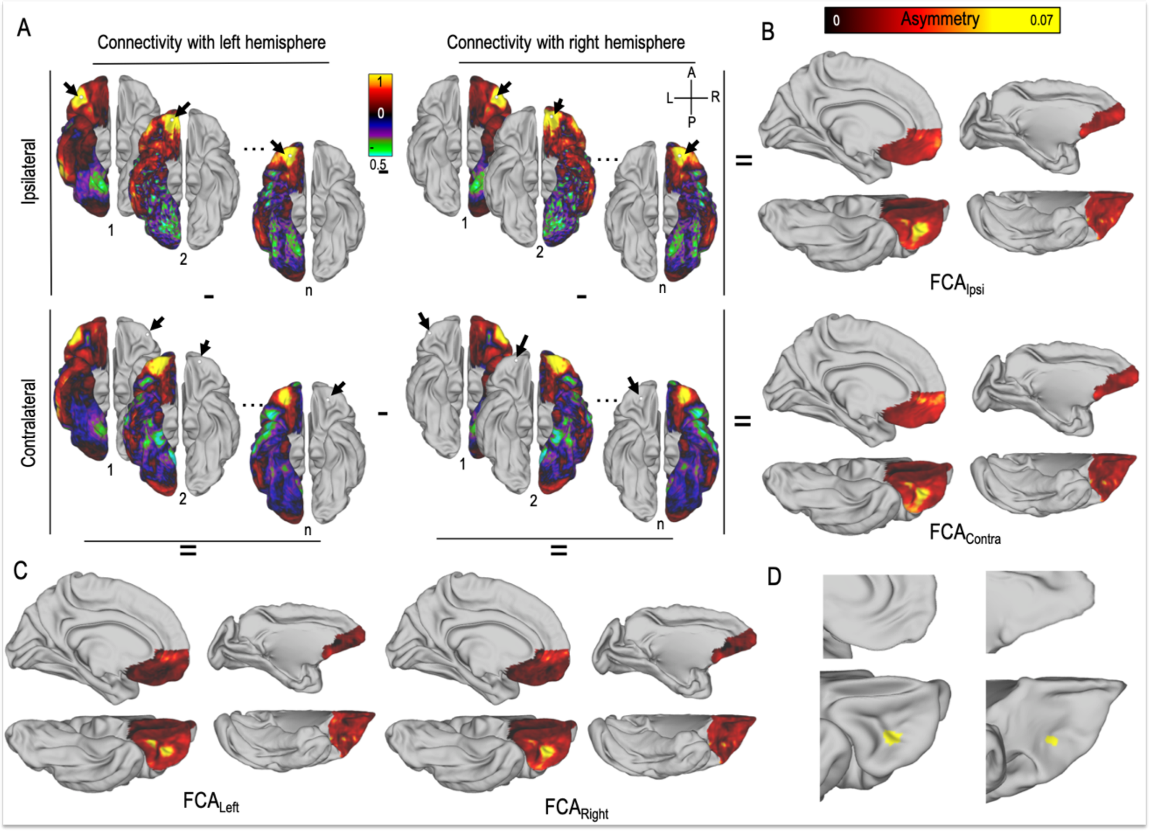

Figure S5 - Functional connectivity asymmetry in the human and macaque OMPFC
A. Schematic representation of the method to compute FCA measures. **Top row**. Ipsilateral frame. The unsigned difference between the functional connectivity of each vertex in the left OMPFC with all vertices in the left hemisphere and the functional connectivity of each vertex in the right OMPFC with all vertices in the right hemisphere is computed. The **left (right) columns** display results for the left (right) hemisphere respectively. Arrows represent the location of seeds while n is the number of vertices in the OMPFC. Colors indicate correlation coefficient between timeseries of the seed and timeseries of each other vertex. **Bottom row**. Contralateral frame. Same as top except that the difference in connectivity is based on the contralateral connectivity of the left and right OMPFC. **B**. The results of these two comparisons between the left and right hemispheres, within the ipsilateral frame (FCA_{Intra}) and the contralateral frame (FCA_{Contr}) are displayed in the **top row** and **bottom row** for humans (**left**) and macaques (**right**). (**C**) The maps resulting from comparison of left and right OMPFC connectivity with the left (FCA_{Left}) and right (FCA_{Right}) hemispheres regardless of whether the hemisphere is contralateral or ipsilateral to the OMPFC region examined. Again humans are shown on the **left** and macaques are shown on the **right**. . Hot colors in B and C indicate high asymmetry in functional connectivity. (**D**) Each map in B and C was then z-scored, thresholded (z>2.3), and clusters surviving correction for multiple comparisons were overlapped in humans (left) and macaques (right). Conjunction analyses of the 4 measures of asymmetry revealed the same cluster of functional asymmetry. In panels B, C, and D results are summarized on left surfaces: A, L, R, P corresponds to Anterior, Left, Right, Posterior respectively.

Fig. S6.

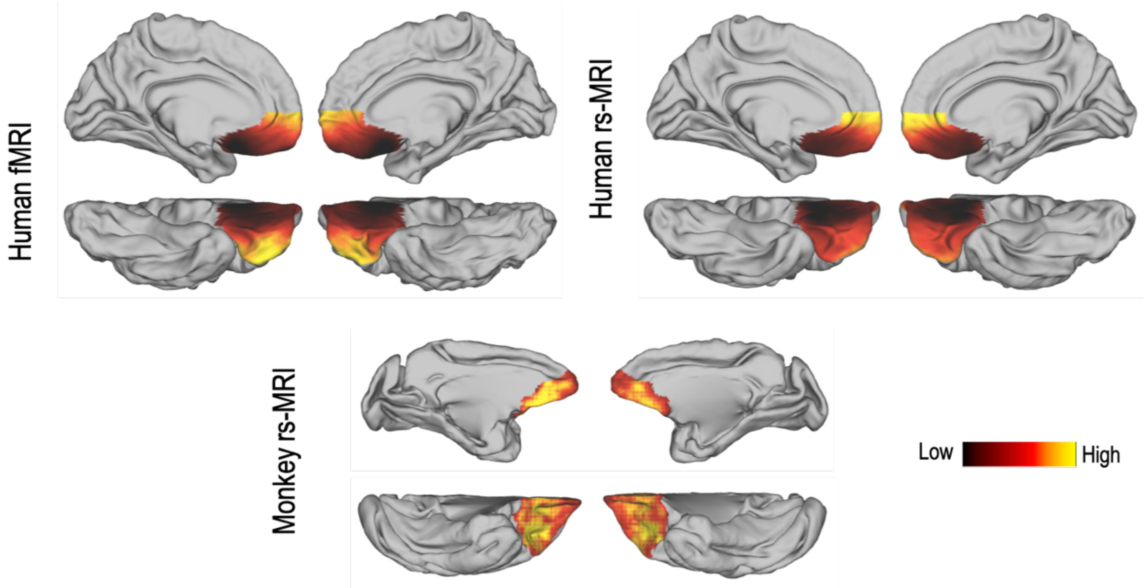

Figure S6. Signal-to-noise ratio (SNR) in fMRI and rs-MRI in the human and macaque OMPFC. Medial and ventral view of human (top) and macaque (bottom) brain. Hot colors indicate high SNR.

Table S1. General Linear Models (GLMs) used in macaque protocols

	Type of event	Stimulus		Decision		Outcome		Intercept	Error
	Type of regressor	Categorical	Parametric	Categorical	Parametric	Categorical	Parametric		
**Protocol 1**	Regressors			DECISION	chosen value	**Reward**	Reward trace	$\beta 0$	ε
					unchosen value				
					unpresented option value				
					choice trace : chosen - unchosen				
					choice trace : unpresented value				
					Choice location				
				right response		**No reward**	Reward trace		
				left response					
				right response (unconvoluted)					
				left response (unconvoluted)					
**Protocol 2 & 3**	Regressors	STIM	**Expected reward (3 levels)**	DECISION	time-to-act on the current trial	REWARD	**Reward amount (4 levels)**	$\beta 0$	ε
			dot speed (3 levels)						
			ITI (3 levels)	right response		distortion due to mouth movements (unconvoluted)			
			reward on previous trial (4 levels)	left response					
			time-to-act on the previous trial	right response (unconvoluted)					
			time from beginning of session	left response (unconvoluted)					
**Protocol 4**	Regressors			DECISION	**Expected reward (3 levels)**	REWARD	**Reward amount (3 levels)**	$\beta 0$	ε
					ResponseTime		reward thrown (3 levels)		
					expected reward thrown (3 levels)		RPE (reward prediction error)		
				Missed Decision			RPE thrown		
				right response		distortion due to mouth movements (unconvoluted)			
				left response					
				right response (unconvoluted)					
				left response (unconvoluted)					

Description of the categorical and parametric regressors used in the GLMs of each macaque protocol to explain BOLD signal variations. Regressors highlighted in green correspond to the ones used in the present study. Right and left response corresponds to left and right sensor press (usually with left and right hand respectively although some macaques occasionally used the same hand for touching both sensors).
